# Assessing Lifestyle Behaviours of People Living with Neurological Conditions: A Panoramic View of Community Dwelling Australians from 2007–2018

**DOI:** 10.3390/jpm11020144

**Published:** 2021-02-19

**Authors:** Nupur Nag, Xin Lin, Maggie Yu, Steve Simpson-Yap, George A. Jelinek, Sandra L. Neate, Michele Levin

**Affiliations:** 1 Neuroepidemiology Unit, Centre of Epidemiology and Biostatistics, Melbourne School of Population and Global Health, The University of Melbourne, Parkville, VIC 3010, Australia; xin.lin2@unimelb.edu.au (X.L.); maggie.yu@unimelb.edu.au (M.Y.); steve.simpsonyap@unimelb.edu.au (S.S.-Y.); g.jelinek@unimelb.edu.au (G.A.J.); sandra.neate@unimelb.edu.au (S.L.N.); 2 Menzies Institute for Medical Research, University of Tasmania, Hobart, TAS 7000, Australia; 3 Roy Morgan Research Institute, Tonic House, Melbourne, VIC 3000, Australia; michele.levine@roymorgan.com

**Keywords:** lifestyle behaviours, diet, cross-sectional, population study, neurological disorders

## Abstract

Neurological disorders pose a substantial health and economic burden to the individual and society, necessitating strategies for effective prevention and disease management. Lifestyle behaviours play a role in risk and management of some neurological disorders; however, overlap between lifestyle behaviours across disorders has not been well explored. We used log-binomial regression to assess associations of selected lifestyle behaviours in community-dwelling Australians (*n* = 192,091), some of whom self-reported Alzheimer’s disease (AD), motor neurone disease (MND), multiple sclerosis (MS), Parkinson’s disease (PD) or stroke. Of six lifestyle behaviours, undertaking physical activity was inversely associated with the presence of all neurological disorders except PD. Smoking was positively associated with MND and stroke, and inversely associated with PD. Participants with AD and stroke shared inverse associations with cognitive engagement, face-to-face social interaction and stress-reducing activities, and MS was positively associated with online social interaction and stress-reduction activities. Of eleven food and beverage consumption categories, no associations were seen in MND, ten categories were inversely associated with people with AD or stroke, and six of these with PD. Vegetable and soft drink consumption were associated with MS. Further detailed assessment of commonalities in lifestyle behaviours across neurological disorders may inform potential strategies for risk reduction across disorders.

## 1. Introduction

The overall burden of neurological disorders continues to increase with an aging population. In 2017, it was estimated that 43% of the Australian population had been diagnosed with a neurological disorder, among these were commonly stroke, Alzheimer’s disease (AD) and dementia, and less commonly motor neurone disease (MND), multiple sclerosis (MS) and Parkinson’s disease (PD) [1]. These disorders present with a heterogenous array of symptoms including cognitive, psychological and physical impairments, which contribute to reduced quality of life for the individual and pose significant societal and economic burden [2,3,4]. These current and increasing burdens necessitate the identification of effective and targeted strategies to achieve risk reduction, manage symptoms, and delay progression.

Modifiable lifestyle behaviours, including diet, physical activity, smoking, cognitive reserve and social interaction have been implicated in the onset and progression of some neurological disorders [5,6,7]. Diets high in saturated fats, including red meat and processed foods, have been associated with increased risk of AD, PD, MS and stroke; while high fruit, vegetable and whole grain intake have been associated with reduced risk [8,9,10,11]. Physical activity has been shown to have benefits for healthy aging and neuroplasticity [12,13], with a minimum of 150 min/week of moderate-intense activity being the international recommendation for adults in maintaining a healthy lifestyle [14,15]. Smoking is similarly well-established as a risk factor for dementia, stroke and MS [6,16,17]; its role for PD risk remains debatable with some studies showing a protective effect [5,18]. Cognitive reserve, enhanced by engaging in physical, leisure and intellectually stimulating activities, may be a mechanism for protection against cognitive and functional decline in the presence of brain pathology [6]. Increasing social interaction and reducing stress are also lifestyle recommendations for optimal brain health [19,20].

The evidence for the role of lifestyle behaviours in risk and management of neurological disorders continues to grow, likely acting concurrently for optimal benefits. Indeed, multimodal lifestyle behaviours, combining healthy diet, increased exercise and cognitive training, have shown improved cognitive outcomes in at-risk elderly people and people with MS [21,22] and a reduced risk of secondary stroke and AD [23,24], suggesting a multi-dimensional approach may be beneficial across different disorders.

Despite the evident role of lifestyle in the prevention and management of different neurological disorders, the epidemiological landscape of lifestyle associations across disorders remains under-examined. Herein, we describe and compare the distributions of modifiable lifestyle behaviours in community-dwelling Australians with and without one of five neurological conditions—AD, PD, MND, MS and stroke. In doing so, we aim to identify shared lifestyle profiles of people with these conditions, which may in turn shed new light on targeted risk reduction and effective self-management strategies.

## 2. Materials and Methods

### 2.1. Study Design and Participants

Each year, 50,000 randomly selected Australian households in 11 major geographic regions are sampled by Roy Morgan Research Institute [25]. The youngest consenting English-speaking member of the household, aged ≥14 years, is interviewed face-to-face by a trained professional. During the interview, the establishment survey including participant demographics is completed and entered into a secure database. Interviewees are then provided with a hard copy of the Single Source Questionnaire (SSQ) for self-completion and asked to return it by post within 30 days to Roy Morgan Research Institutes’s Head Office. Completion is incentivised by entry to a monthly monetary draw prize valued at $1000.

The SSQ comprises a 112-page survey, with 10 sections on various consumer behaviours including interests and attitudes, health conditions, lifestyle and purchasing behaviours, and service and technology utilisation. Data from SSQ surveys are scanned and cleaned for analysis on a quarterly cycle.

### 2.2. Data Collection and Measurement

Participants provided informed consent to Roy Morgan Research Institute for their data to be used for research purposes. The current study was approved by The University of Melbourne, Melbourne School of Population and Global Health Human Ethics Advisory Group, project #1953821.1.

Data extracted includes de-identified adults aged ≥18 years that were interviewed from January 2007–September 2018. SSQ non-responder versus responder biases were analysed based on demographics queried in the face-to-face interview. For main analyses, data inclusion was limited to SSQ responders, and data was extracted on demographics, self-reported neurological disorders and researcher-defined lifestyle behaviours using select variables captured in the SSQ (Table 1).

#### 2.2.1. Classification of Neurological Disorders

Neurological disorders were based on a self-reported tick-box selection of 21 brain and nervous system conditions within 20 condition categories from the section “About your Health”. Data were restricted to those reported for ’You’, in response to the question “Which of the following illnesses or conditions have you or any other member of your household had in the last 12 months?”. Five of 21 listed brain and nervous system conditions were selected as outcome variables: AD, MND, MS, PD and stroke.

Sixteen other SSQ-defined nervous system conditions comprised nine specific neurological conditions (cerebral palsy, chronic fatigue syndrome, epilepsy, nerve damage, neuralgia, neuritis, neuropathy, spinal stenosis, mini stroke) and seven non-specific conditions (face pain, fibromyalgia, frequent headaches, memory problems, meningitis, migraine headaches, tingling sensations).

Participants reporting having more than one of the five conditions of interest (*N* = 58), and those reporting the other nine specific conditions (*N* = 722) were excluded from analysis to allow specificity of the outcome and clarity of signal. The seven non-specific conditions were included in both the comparator population (CP) and neurological disorders of interest populations.

The CP were thus participants who had not self-reported having AD, MND, MS, PD, stroke or any of the nine specific neurological conditions.

#### 2.2.2. Demographics

Demographic variables were categorised as follows: age into tertile year range; BMI according to Word Health Organisation definitions [26]; country of birth from a selection of 13 options: Australia or New Zealand (NZ), Europe, Asia or other (North America, Central and South America, South Pacific, Middle East, Africa, other); religion from a selection of 18 tick-box selection options including ‘No religion’ dichotomised as No/Yes; education dichotomised to No/Yes for the completion of a university degree; employment status as employed (full and part-time), unemployed, student/home duties, and retired; income aligned with Australian Taxation Office taxable income bracket [27]; remoteness based on postal codes; relationship status dichotomised to partnered (married, de facto, engaged, planning to marry) vs. not partnered (single, separated, divorced, widower); and living status dichotomised to lives with others (partner with/without children, single parent, with parents, boarder, shared household) vs. alone (living alone).

#### 2.2.3. Lifestyle Behaviours

Selected SSQ variables were categorised to lifestyle behaviours identified as being associated with neuronal health in the literature (Table 1). These were then dichotimised (No/Yes) for regression analyses.

#### 2.2.4. Food and Beverage Consumption

Food consumption was based on response to “Which of the following have you eaten in the last 7 days”, self-reported tick-box selection on 74 single items within six food categories from the section “Food and Beverages”. Selected food items were recategorised into seven groups (Table 1). Reported serves per day of fruit and vegetables were categorised as per Australian recommended daily serves of ≥2 and ≥5, respectively [28].

Beverage consumption was based on the response to “Consumed in the last 7 days”, self-reported tick-box selection on 36 single items, from which soft drinks, tea/coffee, and milk (included with dairy foods) were included for analysis (Table 1).

Alcohol consumption was based on the response to “Brands drunk in the last 7 days”, self-reported tick-box selection of brand for beer (*n* = 146), cider (*n* = 14), spirits (*n* = 93), and wine (bottled, cask, fortified, sparkling) and “other”; other were excluded from the analysis.

### 2.3. Statistical Analysis

SSQ responder bias was assessed using log-binomial regression [29]. Given the large sample size, reliance on statistical significance as a marker of differences resulted in every association being significantly different. We therefore utilised a crude cut-point of a >50% difference as a benchmark for material and meaningful differences, to inform the development of multivariable models for the primary associations of interest.

Characteristics of having one of the five neurological disorders as compared to the CP, were evaluated by log-binomial regression. Multivariable models were adjusted for age, sex and education, these model covariates having been selected on the basis of the literature review and a priori reasoning.

## 3. Results

### 3.1. Characteristics of SSQ Non-Responders and Responders

Of participants interviewed from January 2007 to September 2018 (*n* = 537,327), 36% (*n* = 192,091) returned the SSQ and were aged ≥18 years (Table 2).

Compared to non-responders, SSQ responders were more likely to be aged ≥40 years (PR _40–59_ = 1.56; PR _≥60_ = 1.84) than 18–39 years. Sex, country of birth, university education, employment status, household income, remoteness, partnered and living status, alcohol consumption and smoking status did not differ more than 50% between the two groups.

### 3.2. Demographics of Analysis Cohort

Participants with AD, PD or stroke were less likely to be female, and they were more likely to be female for MS (PR_AD_ = 0.48; PR_MS_= 2.56; PR_PD_ = 0.59; PR_stroke_ = 0.56; Table 3). Participants with AD, MND, PD or stroke were more likely to be ≥60 years, while participants with MS were more likely to be aged 40–59 years. Participants with stroke were 29% more likely to be obese than the CP. Further, those with AD or stroke were 39% and 36% less likely to be university educated, respectively. Compared to CP, participants with any of the five neurological disorders were more likely to be unemployed or retired. Participants with MND or stroke were less likely to be partnered (PR_MND_ = 0.50; PR_stroke_ = 0.62) and those with MND or stroke were less likely to live with others (PR_MND_ = 0.43; PR_stroke_ = 0.68). Other characteristics are shown in Table 3.

#### 3.2.1. Lifestyle Associations with Neurological Conditions

Participants with AD or stroke were 52% and 55% less likely to undertake cognitively engaging activities than the CP (Table 4). Participants with either AD, MND, MS or stroke were less likely to undertake physical activity (PR_AD_ = 0.50; PR_MND_ = 0.61; PR_MS_ = 0.72; PR_stroke_ = 0.69). Participants with MND or stroke were 2.1 and 1.5 times more likely to be current smokers than the CP, whereas those with PD were 43% less likely. Participants with AD or stroke were 62% and 55% less likely to socialise face-to-face, and those with MS were 68% more likely to socialise online. Participants with AD or stroke were 38% and 24% less likely to engage in stress-reducing activities than the CP, respectively, those with MS were 22% more likely.

#### 3.2.2. Food and Beverage Associations with Neurological Disorders

Participants with AD were 55% less likely to consume bakery/cereals, 52% less likely to consume dairy, and 31% less likely to eat fish/seafood. Similar associations were observed among participants with stroke (Table 5).

Participants with AD, PD or stroke were less likely to consume fruit (PR_AD_ = 0.54; PR_PD_ = 0.78; PR_stroke_ = 0.61) or vegetables (PR_AD_ = 0.41; PR_PD_ = 0.71; PR_stroke_ = 0.56). Of participants who consumed fruit in the past 7 days, recommended daily serves were 21% less likely met by participants with stroke. Those with MS were 42% more likely to eat vegetables. Compared to the CP, participants with AD were 61% less likely to consume meat and almost half as likely to eat natural grains and snacks. Participants with PD or stroke were also less likely to consume those foods.

For beverages consumed in the preceding 7 days, participants with AD, PD and stroke were 39%, 33% and 51% less likely to consume alcohol, respectively, than the CP. Soft drinks were 26% less likely to be consumed by participants with MS and tea/coffee were 47% and 33% less likely to be consumed by those with AD or stroke, respectively.

## 4. Discussion

Understanding the overlap in lifestyle behaviours across neurological disorders provides important information on which to potentially base public health interventions and targeted self-management strategies for potential reduced risk as well as improved health. Cross-sectional population data collected annually over 11 years, from community-dwelling Australians, were pooled to assess associations between lifestyle behaviours and five neurological disorders. Undertaking physical activity was inversely associated with all neurological disorders, except PD. Participants with AD and stroke shared inverse associations across four of six lifestyle behaviours and ten of eleven food and beverage consumed categories. Six food and beverage consumed categories were additionally inversely associated with PD. Few associations were found with participants with MS and MND.

Sociodemographic characteristics were generally as expected, with participants with AD, PD and stroke being older males, and with MS more likely to be 40–59-year-old females. Across disorders, similarities were noted in being unemployed or retired, as well as income range, possibly attributable to older age and/or disability common to these disorders. Participants with MND or stroke were less likely to be partnered, and those with AD or stroke less likely to be educated. These and other demographic associations may assist in identifying resources and services required to provide appropriate support and care.

Physical activity was the only lifestyle behaviour shared across all neurological disorders, except PD, being inversely associated. While this aligns with the lack of physical activity undertaken by people with neurological disorders [6,30], it contradicts findings showing a protective impact on PD [31]. Our findings may be attributed to disease-associated disability, or limitations in data collection which queried physical activity broadly as undertaking either formal exercise or sport.

Inverse associations with ten food and beverage consumed categories were observed in participants with AD and stroke, and in participants with PD, with fruit, vegetable, meat, grains, snacks, and alcohol consumed. These findings may reflect the non-specific mode of assessing dietary intake and differential responding between cases and controls. Alternatively, they may reflect the concept of a role of the microbiota-gut-brain-axis in neurodegenerative disorders, whereby the dysregulation of intestinal microbiota through unbalanced nutrition, antibiotics, age, and infection may lead to pathological processes initiating in the gut and then spreading to the brain via the vagus nerve or circulatory system [32,33].

The importance of measuring quantities consumed is evident in associations persisting only between participants with stroke and fruit consumed, when the number of daily serves were assessed. Participants with MS were more and less likely to consume vegetables and soft drinks, respectively, perhaps reflective of a younger demographic’s attitudes of a healthy diet. The data captured precludes the ability to determine whether food and beverage consumption and avoidance were adopted after disease diagnosis on the basis of medical recommendations or self-management of symptoms. The shared inverse associations of food and beverages with AD, PD and stroke warrant longitudinal investigations employing a research-focused survey using validated tools for assessing foods consumed that capture serve quantities in addition.

Smoking was positively associated with MND and stroke and inversely associated in PD, aligning with previous studies showing smoking as a strong risk factor for MND and stroke and possibly neuroprotective for PD [34,35,36]. Although it is an established risk factor [17], we did not find an association between smoking and MS; it may be that the association is weak or dependent on interaction with other risk factors, or due to responders giving up smoking post-diagnosis.

Cognitive engagement, face-to-face social interaction, and undertaking hobbies as a proxy for stress-reducing activities, were inversely associated with AD and stroke. These findings support the concept of cognitive reserve, enhanced by participating in intellectually stimulating activities, in delaying or preventing cognitive decline in the presence of neuropathology [37]. Simultaneously, these activities may be considered stress-reducing, attenuating the impact of psychological stress on the risk of dementia and stroke [38,39]. In participants with MS, positive associations were seen with online social interaction and stress-reduction, likely reflective of the younger and majority female population; both demographics more likely to engage in such activities [40]. People with MS also have high usage of social media for health information and social support [41].

Overall, physical activity was the only shared lifestyle behaviour of AD, MS, MND and stroke. Interventions for regular exercise and sports may therefore effectively impact multiple neurological disorders. Our findings showed inverse associations with all lifestyle behaviours for both AD and stroke, aligning with the latter being an established risk factor for all-cause dementia [42], and the recognized coincidence of cerebrovascular disease and AD [43]. Alternatively, the similarities may be due to the classification of AD in cases of vascular dementia.

The associations between MS and lifestyle factors were different from other neurological disorders. This may be due to differences in demographic characteristics, cause, or symptoms among these disorders, or under-representation of people with MS in the cohort given SSQ responders comprised lower proportions of participants aged 18–39 years, the age range in which MS is usually diagnosed. MND was not associated with most lifestyle factors except physical activity and smoking.

Limitations are acknowledged and include SSQ responders representing only 36% of participants interviewed over 11 years, to contribute to a market research survey. Responder and non-responder characteristics were mostly similar, albeit responders comprising lower proportions of participants aged 18–39 years. Other limitations include measurement errors inherent with self-reported data, potential under-reporting due to the methodology of data collection, incomplete data, unverified clinical diagnosis, temporality of lifestyle behaviours before and after diagnosis and the co-occurrence of conditions which were excluded for clarity of signal. Despite the limitations, our study has important strengths, primarily extensive data variables from a large community-dwelling population that enable the comparison of numerous characteristics, including a range of lifestyle behaviours and food and beverage consumption, across neurological disorders, which may help direct future research in this area.

## 5. Conclusions and Implementation

The study identified some overlapping associations in lifestyle behaviours and food and beverage consumption in a large community-dwelling population with one of five self-reported neurological disorders. These analyses should be extended to include a comprehensive assessment of the impact of lifestyle behaviours on health outcomes in people with a neurological disorder, to better understand the overlapping role of lifestyle in risk minimisation and disease management.

Cross-sectional studies cannot assess the temporality between modifiable behav-iours and neurological disorders; however, they are important in exploring dis-ease-related characteristics that may inform the planning and allocation of health and research resources. Understanding overlapping relationships across similar disorders may facilitate appropriate and targeted risk reduction and improved self-management through non-invasive and cost-effective methods. Current relationships between life-style factors and neurological disorders need to be further assessed in longitudinal studies to allow the development of effective health interventions in this area.

## Figures and Tables

**Table 1:** Single Source Questionnaire (SSQ) variable inclusions for lifestyle behaviours and food and beverage categories.

Lifestyle	SSQ Variables Queried as Done in the Last 3 Months (Used in Last 4 Weeks, Communication Apps)
Cognitive engagement	Went to a short course/seminar/convention/public lecture; read a novel; read a non-fiction book; used a computer at home; used a computer at work or school; played a musical instrument or sung in a choir; worked on a car; dressmaking
Physical activity	Did some formal exercise; played a sport
Smoker	Current
Social face-to-face	Visited friends/relatives; entertained friends/relatives; held a dinner party
Social online	Facebook Messenger, Skype, Viber, WeChat, WhatsApp, teleconference, telephone
Stress reduction	Hobbies
**Food**	**SSQ Variables Queried as Consumed in the Last 7 days**
Bakery/cereal	Rolls/bread, porridge, cereals (biscuit, other), toast, bagels
Dairy	Milk (from drinks: white, UHT, flavoured, breakfast), yoghurt (natural, flavoured, drinking), cheese (natural, dip), dairy desserts, ice cream (single, tub)
Fish/seafood	Fish, other seafood
Fruit/vegetables	Fresh, canned, frozen, dried
Meat	Chicken, beef, veal, lamb/mutton, pork, turkey, duck, rabbit, ham/bacon, other cold meats, other meats
Natural grains	Rice, pasta/spaghetti, noodles
Snacks	Pastries, muffins/doughnuts, croissants, biscuits (all), chips, muesli bars, breakfast bars, chocolate (all), lollies/mints/gum, frozen desserts, other snacks
**Beverage**	**SSQ Variables Queried as Consumed in the Last 7 days**
Alcohol	Beer, wine, cider, spirits
Soft drinks	Cola, lemonade, lemon, orange, other soft drinks (diet and regular), mixers.
Tea/coffee	Tea, coffee (hot and cold)

**Table 2:** Characteristics of single source survey non-responders and responders.

Characteristic	Non-Responder	Responder	PR
	(*N* = 345,236)	(*N* = 192,091)	(95% CI)
**Sex**			
Male	180.2k (68.7%)	82.0k (31.3%)	1.00
Female	165.0k (60.0%)	110.1k (40.0%)	1.28 (1.27, 1.29)
**Age, years**
18–39	131.9k (75.8%)	42.2k (24.2%)	1.00
40–59	111.2k (62.2%)	67.5k (37.8%)	**1.56 (1.54, 1.57)**
≥60	102.1k (55.3%)	82.4k (44.7%)	**1.84 (1.83, 1.86)**
**Country of birth**
Australia/NZ	251.0k (62.3%)	151.8k (37.7%)	1.00
Europe	40.4k (61.6%)	25.2k (38.4%)	1.02 (1.01, 1.03)
Asia	31.0k (79.5%)	8.0k (20.5%)	0.54 (0.53, 0.55)
Other	22.9k (76.4%)	7.0k (23.6%)	0.63 (0.61, 0.64)
**University education**			
No	217.3k (63.0%)	127.6k (37.0%)	1.00
Yes	127.9k (66.5%)	64.5k (33.5%)	0.91 (0.90, 0.91)
**Employment status**			
Employed	205.7k (68.3%)	95.6k (31.7%)	1.00
Unemployed	29.9k (66.8%)	14.8k (33.2%)	1.05 (1.03, 1.06)
Student/Home Duties	29.5k (66.0%)	15.2k (34.1%)	1.07 (1.06, 1.09)
Retired	80.2k (54.7%)	66.5k (45.3%)	1.42 (1.42, 1.44)
**Income, AUD**			
0–19,999	114.3k (62.2%)	69.5k (37.8%)	1.00
20,000–39,999	86.4k (62.3%)	52.4k (37.7%)	1.00 (0.99, 1.01)
40,000–89,999	103.9k (66.1%)	53.3k (33.9%)	0.90 (0.89, 0.91)
≥90,000	40.4k (70.5%)	16.9k (29.5%)	0.78 (0.77, 0.79)
(Missing)	(202; 80.5%)	(49; 19.5%)	n/a
**Remoteness**			
Capital city	209.7k (66.2%)	106.9k (33.8%)	1.00
Regional	135.6k (61.4%)	85.2k (38.6%)	1.14 (1.13, 1.15)
**Partnered**			
No	146.9k (67.3%)	71.4k (32.7%)	1.00
Yes	198.3k (62.2%)	120.7k (37.8%)	1.16 (1.15, 1.17)
**Lives with others**			
No	69.5k (62.0%)	42.6k (38.0%)	1.00
Yes	272.5k (64.8%)	147.8k (35.2%)	0.93 (0.92, 0.93)
(Missing)	(3.3k; 66.1%)	(1.7k; 34.0%)	n/a
**Alcohol consumption, past 7 days**			
No	144.7k (64.6%)	79.4k (35.4%)	1.00
Yes	200.6k (64.0%)	112.7k (36.0%)	1.02 (1.01, 1.02)
**Current smoker**			
No	271.8k (62.6%)	162.2k (37.4%)	1.00
Yes	73.4k (71.1%)	29.9k (28.9%)	0.77 (0.77, 0.78)

Sample sizes were in the unit of thousands (k). Analysis performed using log-binomial regression models. Shown in bold, PR < 0.50 and >1.50 were used as thresholds for material difference between non-responders and responders.

**Table 3:** Characteristics of neurological disorders referenced to comparator population.

Characteristic	AD		MND		MS		PD		Stroke	
	(*N* = 125)		(*N* = 72)		(*N* = 441)		(*N* = 415)		(*N* = 647)	
	PR	95%CI	PR	95%CI	PR	95%CI	PR	95%CI	PR	95%CI
**Sex**										
Men	Ref		Ref		Ref		Ref		Ref	
Women	**0.48**	**(0.33,0.69)**	1.03	(0.65,1.64)	**2.56**	**(2.04,3.20)**	**0.59**	**(0.48,0.71)**	**0.56**	**(0.48,0.66)**
	***P***	**<0.001**	*P*	=0.91	***P***	**<0.001**	***P***	**<0.001**	***P***	**<0.001**
**Age**										
18–39	Ref		Ref		Ref		Ref		Ref	
40–59	0.70	(0.30,1.60)	0.70	(0.33,1.51)	**2.61**	**(1.97,3.46)**	**7.09**	**(2.55,19.73)**	**5.54**	**(3.25,9.43)**
≥60	**4.58**	**(2.43,8.62)**	**1.93**	**(1.05,3.55)**	1.26	(0.93,1.72)	**44.79**	**(16.62,120.68)**	**14.69**	**(8.78,24.58)**
	**PTREND**	**<0.001**	**PTREND**	**=0.034**	PTREND	=0.14	**PTREND**	**<0.001**	**PTREND**	**<0.001**
**BMI**										
Under/normal	Ref		Ref		Ref		Ref		Ref	
Overweight	1.03	(0.67,1.59)	1.49	(0.84,2.64)	0.97	(0.77,1.23)	1.06	(0.84,1.34)	0.93	(0.76,1.13)
Obese	1.01	(0.63,1.62)	1.07	(0.56,2.02)	1.08	(0.85,1.36)	0.96	(0.74,1.24)	**1.29**	**(1.06,1.57)**
	PTREND	=0.97	PTREND	=0.79	PTREND	=0.55	PTREND	=0.77	**PTREND**	**=0.011**
**Country of birth**										
Australia/NZ	Ref		Ref		Ref		Ref		Ref	
Europe	1.04	(0.65,1.67)	1.77	(0.99,3.15)	1.18	(0.90,1.53)	0.81	(0.62,1.06)	0.94	(0.76,1.17)
Asia	**3.75**	**(1.93,7.30)**	1.31	(0.37,4.56)	**0.18**	**(0.06,0.55)**	**0.51**	**(0.19,1.37)**	1.47	(0.91,2.35)
Other	1.61	(0.67,3.91)	2.32	(0.89,6.07)	**0.42**	**(0.20,0.88)**	0.75	(0.39,1.46)	1.14	(0.73,1.79)
	**PTREND**	**=0.017**	**PTREND**	**=0.031**	**PTREND**	**=0.002**	PTREND	=0.063	PTREND	=0.47
**Religion**										
No	Ref		Ref		Ref		Ref		Ref	
Yes	1.34	(0.85,2.11)	0.67	(0.41,1.09)	0.83	(0.67,1.01)	1.13	(0.89,1.42)	1.03	(0.86,1.23)
	*P*	=0.21	*P*	=0.11	*P*	=0.065	*P*	=0.32	*P*	=0.77
**University education**										
No	Ref		Ref		Ref		Ref		Ref	
Yes	**0.61**	**(0.40,0.95)**	1.28	(0.80,2.05)	1.19	(0.97,1.45)	0.91	(0.72,1.14)	**0.64**	**(0.52,0.78)**
	***P***	**=0.027**	*P*	=0.30	*P*	=0.088	*P*	=0.40	***P***	**<0.001**
**Employment status**										
Employed	Ref		Ref		Ref		Ref		Ref	
Unemployed	**3.89**	**(1.82,8.29)**	**2.70**	**(1.13,6.44)**	**2.86**	**(2.17,3.78)**	**2.93**	**(1.83,4.69)**	**4.46**	**(3.25,6.12)**
Student/home duties	2.30	(0.81,6.48)	**2.52**	**(1.00,6.33)**	1.36	(0.95,1.95)	0.66	(0.24,1.85)	**3.41**	**(2.26,5.15)**
Retired	3.23	(1.78,5.84)	**2.69**	**(1.19,6.07)**	**3.18**	**(2.26,4.48)**	**3.03**	**(2.14,4.29)**	**4.13**	**(3.09,5.52)**
	**PTREND**	**<0.001**	**PTREND**	**=0.009**	**PTREND**	**<0.001**	**PTREND**	**<0.001**	**PTREND**	**<0.001**
**Income, AUD**										
0–19,999	Ref		Ref		Ref		Ref		Ref	
20,000–39,999	0.72	(0.48,1.08)	1.32	(0.79,2.22)	0.85	(0.67,1.06)	0.96	(0.77,1.19)	**0.67**	**(0.56,0.80)**
40,000–89,999	**0.37**	**(0.21,0.66)**	0.58	(0.29,1.16)	**0.60**	**(0.46,0.79)**	**0.65**	**(0.48,0.87)**	**0.34**	**(0.26,0.43)**
>–90,000	**0.17**	**(0.04,0.68)**	0.30	(0.07,1.28)	**0.57**	**(0.37,0.87)**	**0.32**	**(0.17,0.62)**	**0.21**	**(0.12,0.35)**
	**PTREND**	**<0.001**	**PTREND**	**=0.040**	**PTREND**	**<0.001**	**PTREND**	**<0.001**	**PTREND**	**<0.001**
**Remoteness**										
Capital city	Ref		Ref		Ref		Ref		Ref	
Regional	0.88	(0.62,1.24)	0.83	(0.51,1.33)	1.06	(0.87,1.28)	1.07	(0.88,1.29)	1.03	(0.88,1.20)
	*P*	=0.46	*P*	=0.43	*P*	=0.58	*P*	=0.52	*P*	=0.70
**Partnered**										
No	Ref		Ref		Ref		Ref		Ref	
Yes	0.89	(0.61,1.30)	**0.50**	**(0.31,0.81)**	1.00	(0.82,1.22)	0.90	(0.73,1.11)	**0.62**	**(0.53,0.73)**
	*P*	=0.55	***P***	**=0.005**	*P*	=0.98	*P*	=0.34	***P***	**<0.001**
**Lives with others**										
No	Ref		Ref		Ref		Ref		Ref	
Yes	0.90	(0.60,1.35)	**0.43**	**(0.26,0.71)**	0.89	(0.71,1.13)	0.89	(0.71,1.11)	**0.68**	**(0.57,0.81)**
	*P*	=0.62	***P***	**=0.001**	*P*	=0.36	*P*	=0.32	***P***	**<0.001**

Analysis performed using log-binomial regression, adjusted for age, sex and education. Results in boldface denote statistical significance (*p* < 0.05). Abbreviations: AD = Alzheimer’s disease; BMI = body mass index; MND = motor neuron disease; MS = multiple sclerosis; PD = Parkinson’s disease; Ref: reference category; SES = socioeconomic status.

**Table 4:** Associations between lifestyle behaviours and neurological disorders referenced to comparator population.

Lifestyle Behaviour	AD		MND		MS		PD		Stroke	
	(*N* = 125)		(*N* = 72)		(*N* = 441)		(*N* = 415)		(*N* = 647)	
	PR	(95%CI)	PR	(95%CI)	PR	(95%CI)	PR	(95%CI)	PR	(95%CI)
**Cognitive engagement**										
No	Ref		Ref		Ref		Ref		Ref	
Yes	**0.48**	**(0.33,0.69)**	1.03	(0.59,1.80)	0.99	(0.78,1.26)	0.83	(0.67,1.02)	**0.45**	**(0.38,0.53)**
	***P***	**<0.001**	*P*	=0.91	*P*	=0.94	*P*	=0.077	***P***	**<0.001**
**Physical activity**										
No	Ref		Ref		Ref		Ref		Ref	
Yes	**0.50**	**(0.33,0.76)**	**0.61**	**(0.38,0.96)**	**0.72**	**(0.59,0.87)**	0.83	(0.68,1.01)	**0.69**	**(0.58,0.81)**
	***P***	**=0.001**	***P***	**=0.034**	***P***	**<0.001**	*P*	=0.064	***P***	**<0.001**
**Smoker**										
No	Ref		Ref		Ref		Ref		Ref	
Yes	1.07	(0.62,1.82)	**2.14**	**(1.21,3.77)**	1.13	(0.88,1.46)	**0.57**	**(0.39,0.84)**	**1.49**	**(1.22,1.83)**
	*P*	=0.82	***P***	**=0.008**	*P*	=0.34	***P***	**=0.005**	***P***	**<0.001**
**Social face-to-face**										
No	Ref		Ref		Ref		Ref		Ref	
Yes	**0.38**	**(0.26,0.57)**	1.10	(0.53,2.30)	1.10	(0.79,1.52)	0.93	(0.71,1.21)	**0.45**	**(0.37,0.53)**
	***P***	**<0.001**	*P*	=0.80	*P*	=0.57	*P*	=0.58	***P***	**<0.001**
**Social online**										
No	Ref		Ref		Ref		Ref		Ref	
Yes	0.36	(0.11,1.16)	1.39	(0.66,2.91)	**1.68**	**(1.29,2.19)**	1.07	(0.73,1.58)	0.74	(0.51,1.06)
	*P*	=0.088	*P*	=0.39	***P***	**<0.001**	*P*	=0.73	*P*	=0.10
**Stress reduction**										
No	Ref		Ref		Ref		Ref		Ref	
Yes	**0.62**	**(0.40,0.99)**	1.07	(0.65,1.78)	**1.22**	**(1.00,1.48)**	0.88	(0.70,1.10)	**0.76**	**(0.63,0.92)**
	***P***	**=0.043**	*P*	=0.78	***P***	**=0.048**	*P*	=0.25	***P***	**=0.004**

Analysis performed using log-binomial regression, adjusted for age, sex and education. Results in boldface denote statistical significance (*p* < 0.05). Abbreviations: AD = Alzheimer’s disease; MND = motor neuron disease; MS = multiple sclerosis; PD = Parkinson’s disease; Ref: reference category.

**Table 5:** Associations between food and beverage consumption and neurological disorders, referenced to the comparator population.

Food & Beverage	AD		MND		MS		PD		Stroke	
	(*N* = 125)		(*N* = 72)		(*N* = 441)		(*N* = 415)		(*N* = 647)	
	PR	(95%CI)	PR	(95%CI)	PR	(95%CI)	PR	(95%CI)	PR	(95%CI)
Food consumed last 7 days										
**Bakery/cereals**										
No	Ref		Ref		Ref		Ref		Ref	
Yes	**0.45**	**(0.30,0.66)**	1.83	(0.79,4.25)	0.96	(0.74,1.25)	0.85	(0.65,1.11)	**0.60**	**(0.49,0.72)**
	***P***	**<0.001**	*P*	=0.16	*P*	=0.75	*P*	=0.22	***P***	**<0.001**
**Dairy**										
No	Ref		Ref		Ref		Ref		Ref	
Yes	**0.48**	**(0.29,0.77)**	1.53	(0.55,4.25)	0.97	(0.69,1.37)	0.78	(0.57,1.08)	**0.52**	**(0.42,0.65)**
	***P***	**=0.002**	*P*	=0.41	*P*	=0.86	*P*	=0.14	***P***	**<0.001**
**Fish/seafood**										
No	Ref		Ref		Ref		Ref		Ref	
Yes	**0.69**	**(0.48,0.98)**	0.97	(0.60,1.57)	0.87	(0.72,1.06)	1.05	(0.85,1.30)	**0.70**	**(0.60,0.82)**
	***P***	**=0.041**	*P*	=0.92	*P*	=0.17	*P*	=0.64	***P***	**<0.001**
**Fruit**										
No	Ref		Ref		Ref		Ref		Ref	
Yes	**0.54**	**(0.36,0.79)**	1.46	(0.72,2.97)	1.13	(0.87,1.47)	**0.78**	**(0.62,0.99)**	**0.61**	**(0.51,0.72)**
	***P***	**=0.002**	*P*	=0.29	*P*	=0.36	***P***	**=0.044**	***P***	**<0.001**
**Fruit ≥ 2 serve/day**										
No	Ref		Ref		Ref		Ref		Ref	
Yes	1.04	(0.66,1.62)	1.00	(0.57,1.76)	1.20	(0.96,1.51)	1.21	(0.97,1.52)	**0.79**	**(0.64,0.98)**
	*P*	=0.87	*P*	=0.99	*P*	=0.11	*P*	=0.096	***P***	**=0.029**
**Vegetables**										
No	Ref		Ref		Ref		Ref		Ref	
Yes	**0.41**	**(0.27,0.62)**	1.22	(0.56,2.67)	**1.42**	**(1.00,2.02)**	**0.71**	**(0.55,0.92)**	**0.56**	**(0.46,0.68)**
	***P***	**<0.001**	*P*	=0.62	***P***	**=0.048**	***P***	**=0.011**	***P***	**<0.001**
**Veg ≥ 5 serve/day**										
No	Ref		Ref		Ref		Ref		Ref	
Yes	1.22	(0.59,2.49)	0.94	(0.34,2.57)	1.21	(0.83,1.78)	1.23	(0.84,1.80)	1.29	(0.95,1.75)
	*P*	=0.59	*P*	=0.90	*P*	=0.32	*P*	=0.28	*P*	=0.11
**Meat**										
No	Ref		Ref		Ref		Ref		Ref	
Yes	**0.39**	**(0.24,0.61)**	1.51	(0.55,4.13)	0.90	(0.65,1.25)	**0.66**	**(0.48,0.91)**	**0.58**	**(0.46,0.74)**
	***P***	**<0.001**	*P*	=0.42	*P*	=0.53	***P***	**=0.010**	***P***	**<0.001**
**Natural grains**										
No	Ref		Ref		Ref		Ref		Ref	
Yes	**0.51**	**(0.35,0.75)**	1.24	(0.69,2.23)	1.08	(0.85,1.36)	**0.81**	**(0.66,1.00)**	**0.72**	**(0.61,0.85)**
	***P***	**<0.001**	*P*	=0.47	*P*	=0.53	***P***	**=0.047**	***P***	**<0.001**
**Snacks**										
No	Ref		Ref		Ref		Ref		Ref	
Yes	**0.51**	**(0.33,0.77)**	0.98	(0.49,1.95)	0.93	(0.70,1.25)	**0.70**	**(0.55,0.91)**	**0.53**	**(0.44,0.64)**
	***P***	**=0.002**	*P*	=0.95	*P*	=0.64	***P***	**=0.006**	***P***	**<0.001**
Beverages consumed past 7 days										
**Alcohol**										
No	Ref		Ref		Ref		Ref		Ref	
Yes	**0.61**	**(0.43,0.89)**	1.27	(0.78,2.06)	0.86	(0.71,1.04)	**0.67**	**(0.55,0.82)**	**0.49**	**(0.42,0.58)**
	***P***	**=0.009**	*P*	=0.34	*P*	=0.11	***P***	**<0.001**	***P***	**<0.001**
**Soft drinks**										
No	Ref		Ref		Ref		Ref		Ref	
Yes	0.92	(0.62,1.39)	0.99	(0.61,1.62)	**0.74**	**(0.61,0.90)**	1.02	(0.83,1.25)	1.10	(0.93,1.31)
	*P*	=0.70	*P*	=0.97	***P***	**=0.003**	*P*	=0.88	*P*	=0.26
**Tea/coffee**										
No	Ref		Ref		Ref		Ref		Ref	
Yes	**0.53**	**(0.35,0.81)**	1.34	(0.68,2.66)	0.82	(0.64,1.04)	0.80	(0.62,1.02)	**0.67**	**(0.55,0.82)**
	***P***	**=0.004**	*P*	=0.40	*P*	=0.10	*P*	=0.074	***P***	**<0.001**

Analysis performed using log-binomial regression, adjusted for age, sex and education. Results in boldface denote statistical significance (*p* < 0.05). Abbreviations: AD = Alzheimer’s disease; MND =motor neuron disease; MS = multiple sclerosis; PD = Parkinson’s disease; Ref: reference category.

## Data Availability

Restrictions apply to the availability of these data. Data was obtained from Roy Morgan Research Institute and may be requested from M.L. with the permission of Roy Morgan Research Institute.
